# Pharmacological effects of natural medicine ginsenosides against Alzheimer’s disease

**DOI:** 10.3389/fphar.2022.952332

**Published:** 2022-11-16

**Authors:** Zhikun Shi, Hongyu Chen, Xu Zhou, Wei Yang, Yang Lin

**Affiliations:** ^1^ Department of Gynecology and Obstetrics, The Second Hospital of Jilin University, Changchun, China; ^2^ Jilin Provincial Key Laboratory on Molecular and Chemical Genetic, The Second Hospital of Jilin University, Changchun, China

**Keywords:** Alzheimer’s disease, ginseng, ginsenoside, neuroprotective effects, β-amyloid, neurofibrillary tangle

## Abstract

Ginsenosides are the most important pharmacological active ingredient of ginseng, with multiple biological therapeutic targets, mild action and no side effects. It is having shown beneficial effects *in vitro* and *in vivo* models of AD. In this review, we analyze large literature, summarize the inhibition of ginsenosides fibrous extracellular deposition of β-amyloid (Aβ) and neurofibrillary tangles (NFTs) of possible mechanisms, and explain the effects of ginsenosides on AD neuroprotection from the aspects of antioxidant, anti-inflammatory, and anti-apoptosis, prove the potential of ginsenosides as a new class of drugs for the treatment of AD. In addition, according to the current clinical application status of natural drugs, this paper analysis the delivery route and delivery mode of ginsenosides from the perspective of pharmacokinetics, providing a deeper insight into the clinical application of ginsenosides in the treatment of AD.

## Introduction

**FIGURE 1:**
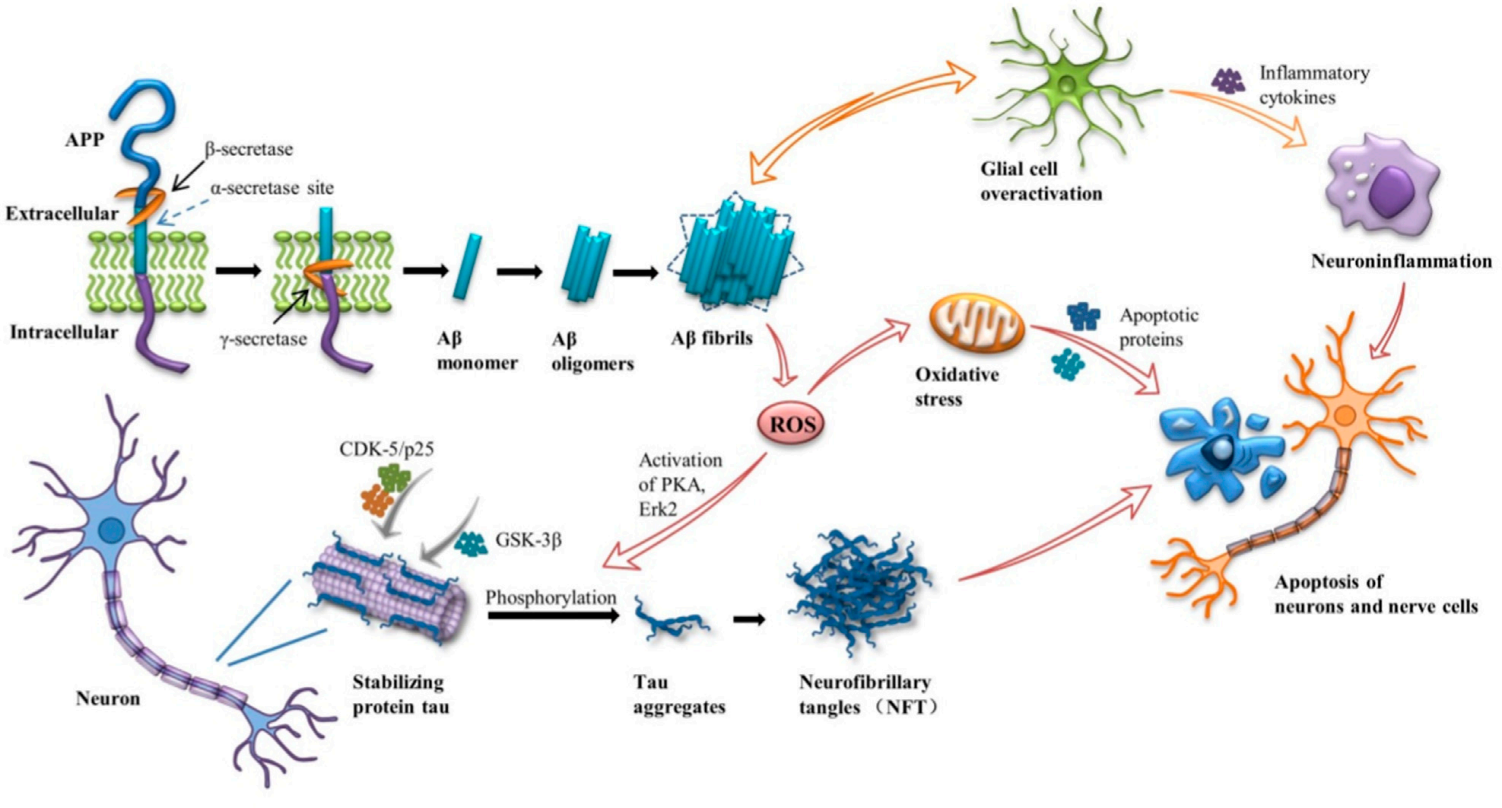
Amyloid cascade in Alzheimer’s disease (AD). APP, amyloid -β protein precursors; CDK-5, cyclin-dependent kinase-5; GSK3-β, glycogen synthase kinase 3-β; PKA, protein kinase A; Erk2, extracellular signal-regulated kinase 2.

Alzheimer’s disease (AD) is a chronic progressive neurodegenerative disease, namely the gradual loss of neuronal structure and function in the brain, characterized by memory loss, cognitive and functional deficits, and behavioral disorders. At present, no effective clinical drugs have been found to prevent the progression of the disease. Though the pathogenesis of the disease is not fully understood, its major pathological features have been identified: extracellular β-amyloid (Aβ) formation and aggregates of the phosphorylated microtubule-associated protein Tau in neurofibrillary tangles (NFTs) (Lane et al., 2018) (Figure 1). More and more evidence suggests that Aβ and tau proteins begin to accumulate years before clinical symptoms appear (Bejanin et al., 2017). According to the amyloid cascade hypothesis, it is the main influencing factor that the accumulation of Aβ in the brain drives the pathogenesis of AD. Continuous aggregation and deposition of Aβ peptides induce inflammation and microglial cascades, broad-spectrum ion and neurotransmitter abnormalities, mitochondrial dysfunction, oxidative stress, etc. The imbalance of Aβ production and clearance leads to the hyperphosphorylation of tau to NFTs, which further leads to synaptic and neuronal dysfunction and destruction, ultimately leading to extensive cortical dysfunction (Atri, 2019). In addition to the Aβ hypothesis, the cholinergic hypothesis has a place in the development of potential therapies for AD. The hypothesis suggests that cholinergic deficits are thought to be responsible for the cognitive, behavioral, and overall functional characteristics of AD. At present, the treatment of neurological diseases is mainly drug and surgical treatment. Current treatment methods mainly use acetylcholinesterase (AChE) inhibitors, such as Donepezil, Rivastigmine hydrogen tartrate, Galanthamine, etc. (Lane et al., 2018), to enhance cholinergic neurotransmission by preventing the hydrolysis of acetylcholine and subsequently increasing its synaptic level. However, the effect is not significant, and long-term use of drugs can lead to drug accumulation and poisoning, resulting in certain side effects, such as vomiting, diarrhea and other gastrointestinal reactions (Vaz and Silvestre, 2020). It is worth noting that these drugs can only improve some of the main symptoms of AD, but do not delay or reverse the onset of AD. On the other hand, surgical treatment often increases the chance of infection and other dysfunction.

**FIGURE 2:**
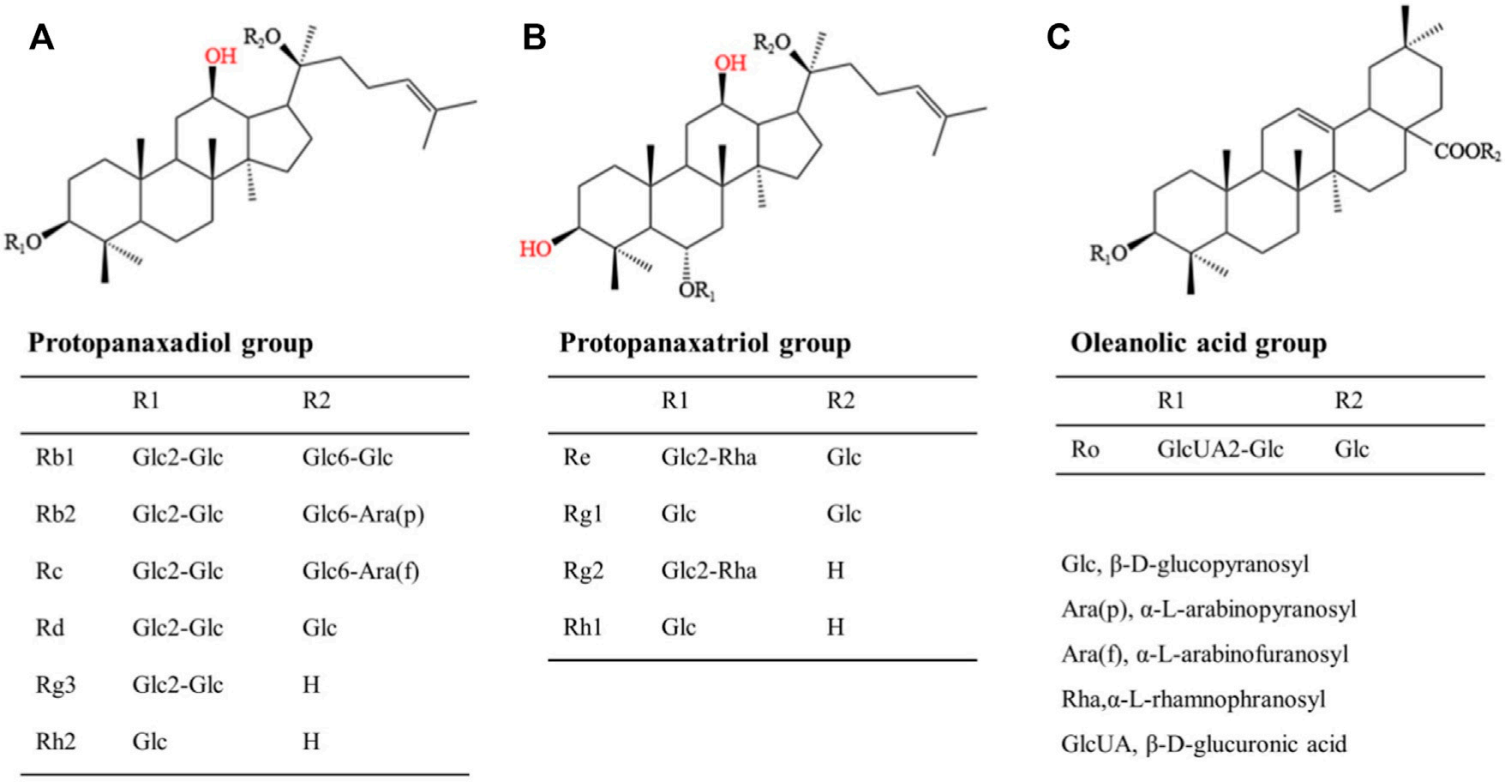
Structural formula of three kinds of ginsenosides. **(A)**: Protopanaxadiol group includes Rb1, Rb2, Rc, Rd, Rg3, Rh2, etc; **(B)**: Protopanaxatriol group includes Re, Rg1, Rg2, Rh1, etc; **(C)**: Oleanolic acid group includes Ro, etc.

**FIGURE 3:**
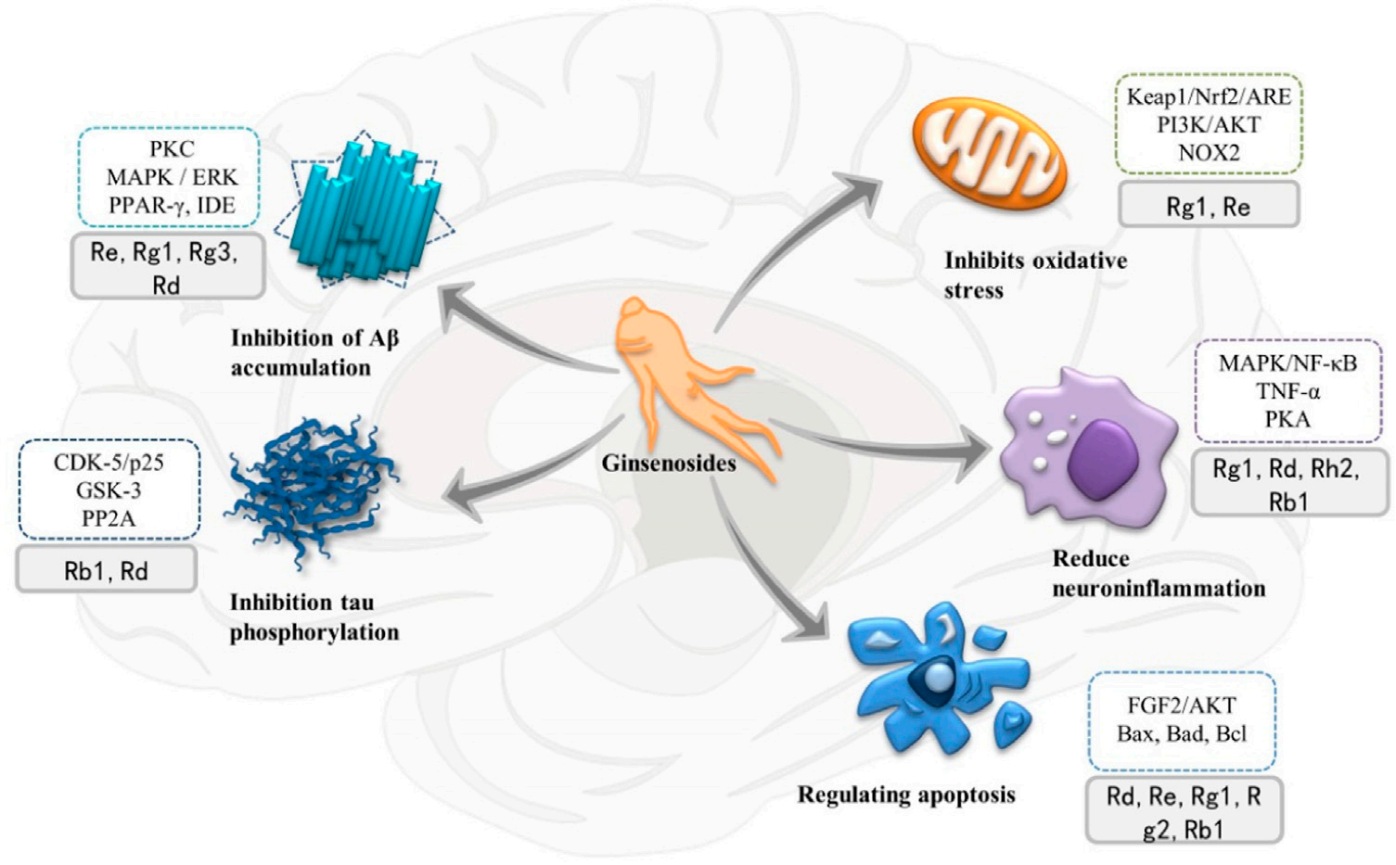
Multiple therapeutic targets of ginsenosides on Alzheimer’s disease (AD) and related signaling pathways and proteins involved. PKC, protein kinase C; MAPK, mitogen-activated protein kinase; ERK, extracellular signal-regulated kinase; PPAR-γ, peroxisome proliferator-activated receptor-γ; IDE, insulin-degrading enzymes; CDK-5, cyclin-dependent kinase-5; GSK3, glycogen synthase kinase 3; PP2A, protein phosphatase-2A; mTOR, mechanized targets of rapamycin; NF-κB, nuclear factor Kappa B; PKA, protein kinase A; TNF-α, Tumor necrosis factor α; PI3K, phosphoinositol 3 kinase.

For the past few years, researchers have increasingly focused their attention on bioactive chemicals in natural medicine for new inspiration and the development of new therapeutic drugs. Ginseng is a kind of popular natural Chinese herbal medicine, including Korean Red Ginseng (KRG), Panax quinquefolius, etc. It is widely used in China, Japan and Korea due to its multiple pharmacological activities and low toxicity and side effect. Its clinical application is extensive, with anti-cancer, anti-inflammatory, antioxidant and vasodilation regulation effects, with great development and utilization value (Dai et al., 2017). In recent years, many molecular targets of ginseng have been identified. Ginsenosides, the first active ingredient isolated from ginseng, can be extracted from root, stem, leaf and fruit, and are mostly distributed in the outer cortex and tubing of the root (Christensen, 2008; Xu et al., 2017). About 30 ginsenosides have been identified from ginseng using existing isolation techniques and processing methods. Ginsenosides Rb1 and Rg1 are the most abundant ginsenosides in ginseng root. High-performance liquid chromatography (HPLC) showed that the content of ginsenoside Rg1 in dried ginseng root was 0.22 ± 0.02% (Xie et al., 2018). Especially, steamed Panax ginseng is the only marketed ginseng known to contain ginsenoside Rg3 (Jovanovski et al., 2014). In terms of structure, ginsenosides all contain the same tetracyclic hydrophobic steroid structure with different sugar components attached. In terms of chemistry, according to the number and position of sugar components, ginsenosides can be divided into protopanaxadiol (PPD) groups, protopanaxatriol (PPT) groups and oleanolic acid groups (Cho, 2012), structure of different ginsenosides types is shown in Figure 2. At present, the identification of ginsenosides structure mainly adopts UV, IR, NMR, and MS modern structure identification techniques combined with physical and chemical properties and elemental analysis methods. Differential thermal analysis, thermogravimetric analysis, powder X-ray diffraction, and other supporting methods can be used to further confirm the structure of ginsenosides. Factors such as substituents, number and configuration of sugar all affect the neuroprotective mechanism of ginsenosides in the AD process (Figure 3). Among them, ginsenosides Rb1, Rb2, Rc, Rd, Re, and Rg1 account for more than 90% of the total ginsenosides of ginseng, and are the most studied at present (Mohanan et al., 2018). Recently, it has been demonstrated that kinds of ginsenosides have beneficial effects in both *in vitro* and *in vivo* models of AD (Table 1). Ginsenosides have different neuropharmacological effects. In addition to inhibiting the formation of Aβ and Tau hyperphosphorylation to form NFTs to delay the process of AD, ginsenosides also exert neuroprotective effects through different mechanisms, including inhibiting oxidative stress, regulating neuroinflammation, improving mitochondrial dysfunction and reducing toxin-induced apoptosis (Kim et al., 2018). For example, ginsenoside Rg1, one of the most widely studied active ingredients in ginseng, has been proved to have antioxidant, anti-inflammatory and anti-apoptotic effects in a large number of *in vivo* and *in vitro* experiments, and can relieve nerve damage and cognitive dysfunction in the process of AD (Long et al., 2014).

**TABLE 1:** Effect of ginsenosides on the pathogenesis of AD.

Model	Drugs	Research results	Authors/Refs.
N2a/APP695 cell line	Ginsenoside Re	Activation PPARγ; Activity BACE1↓; Level Aβ↓	Cao G et al./(Cao et al., 2016)
Tg mAPP mice	Ginsenoside Rg1	Activation γ-secretase ↓; Activation PKA/CREB signaling	Fang F et al./( (Fang et al., 2012))
Male SD rats with injection Aβ1-42	Ginsenoside Rg1	Activation PPARγ; Expression IDE↑; Level Aβ1-42↓; Learning and memory↑	Quan Q et al./(Quan et al., 2013)
AD mice (6-month-old)	Ginsenoside Rg1	Aβ1-42 and (p)-Tau accumulations↓; Activation BDNF-TrkB pathway	Yang X et al./(Yang et al., 2021)
male SD rats (injected with D-gal)	Ginsenoside Rg1	protect NSCs/NPCs ; promote neurogenesis	Zhu J et al./(Zhu et al., 2014)
C57BL/6 mice (injected with D-gal)	Ginsenoside Rg1	Oxidative stress↓; Akt/mTOR signaling pathway↓	Chen L et al./(Chen et al., 2018)
mice (injected with 3-nitropropionic acid)	Ginsenoside Rg1	MAPKs and NF-κΒ signaling pathways↓	Yang X et al./(Yang et al., 2021)
Mice exposed to aluminum chloride	Ginsenoside Rb1	Learning and memory↑; reversing the p-GSK3 and PP2A level	Zhao H et al./(Zhao et al., 2013)
Eighty-four male Sprague-Dawley rats	Ginsenoside Rb1	Expression Bax and caspase-3↓; Expression Bcl-2↑; Inhibit neuronal apoptosis process	Wang Y et al./(Wang et al., 2018)
Wild type and APP/PS1 AD mice	Ginsenoside Rg1	ROS and NOX2 expression↓; APP expression↓; Aβ generation↓; p-Tau level↓;	Zhang H et al./(Zhang et al., 2021)
APP transgenic mice	Ginsenoside Rd	NFκB p65 expression↓; pro-inflammatory factors expression↓;	Liu J et al./(Liu et al., 2015b)
Adult male Sprague-Dawley with injection okadaic acid	Ginsenoside Rd	activities of PP-2A↑; OA-induced the hyperphosphorylation of tau↓;	Li L et al./(Li et al., 2011)

### Inhibitory effect of ginsenosides on Aβ formation

Aβ is the generic name for several peptides composed of amino acid residues cleaved by amyloid -β protein precursors (AβPPs or APP), which are neurotoxic. The amyloid pathway is initiated by β -secretase (BACE1) and γ -secretase, which cleaves APP to produce insoluble Aβ. Accumulation of extracellular Aβ causes it to accumulate in oligomers, protofibrils, and fibrils and eventually deposit as insoluble plaques that damage nearby neurons, leading to synaptic disruption or neuronal death. It was found that Aβ-42 containing 42 amino acid residues was most likely to produce this aggregation (Lane et al., 2018). The non-amyloid pathway produces soluble amyloid precursor protein α (sAPPα), a protein that protects and promotes neuronal proliferation, which is obtained by α -secretase and γ -secretase cleaving the middle portion of the Aβ peptide (Habib et al., 2017). BACE1 is mainly expressed in hippocampal neurons, cerebral cortex and cerebellum granular layer, and its transcription is regulated by a variety of transcription factors, among which the most important is peroxisome proliferator-activated receptor-γ (PPAR-γ), whose activation can inhibit BACE1 promoter activity and thus inhibit Aβ production. In contrast, Sp1 and nuclear factor kappa-B (NF-κB) up-regulate BACE1 activity to promote Aβ production through activation or trans-activation (Christensen et al., 2004; Buggia-Prevot et al., 2008). Fang et al. demonstrated that ginsenoside Rg1 treatment reduced γ-secretase activity and improved cognition and induced neuroprotection by activating protein kinase A/cyclic-AMP response element binding protein (PKA/CREB) signaling in Tg mAPP mice (Fang et al., 2012). Studies have shown that presenilin-KO inhibition of γ-secretase may lead to abnormal development in mice, while BACE1 knockout mice develop normally and Aβ formation is significantly reduced (Luo et al., 2001). Therefore, the current treatment strategies for Aβ peptide in AD mainly include inhibition of β -secretase to inhibit the amyloid pathway and reduce the production of Aβ -peptide, or activation of α -secretase to promote the production of soluble sAPPα (Yan et al., 2017).

Recent studies have demonstrated that some active ingredients in ginseng can inhibit Aβ -induced neurotoxicity and reduce its accumulation in the brain to play an anti-AD role. Molecular docking and *in vitro* studies showed that ginsenoside Rc had the strongest inhibitory effect on BACE1, followed by Rg1, Rb1, Re, etc., (Jovanovski et al., 2014). Ginsenoside Re is the main active ingredient in ginseng. Cao G et al. demonstrated in APP overexpressed neuronal cell model that ginsenoside Re can significantly increase the expression of PPAR-γ, inhibit BACE1 activity, and ultimately reduce the production of Aβ. In addition, the total levels of APP and sAPPα were not affected (Cao et al., 2016). Interestingly, another piece of evidence suggests that Rg1 may act as a PPAR-γ agonist, increasing the binding of nuclear PPAR-γ to the BACE1 promoter, thereby inhibiting BACE1 transcription and translation, inhibiting BACE1 activity, and ultimately reducing amyloid β protein production (Chen et al., 2012). Insulin-degrading enzymes (IDE) is a peptidase that cleaves small proteins of different sequences that facilitate the formation of β-rich amyloid fibrils, primarily to promote insulin catabolism, it can also effectively degrade Aβ in the brain and eliminate the neurotoxic effects of Aβ (Farris et al., 2003). Further studies showed that PPAR-γ promoted gene transcription and expression through binding to functional peroxisome proliferator reaction element (PPRE) in IDE promoter (Du et al., 2009). Quan Q et al. demonstrated that ginsenoside Rg1 can increase IDE expression by up-regulating PPAR-γ, resulting in decreased Aβ levels, alleviating neural damage, and improving learning and memory in depressed rat models (Quan et al., 2013). In addition to IDE, Neprilysin enkephalinase (NEP) is an amyloid protein degrading enzyme that is reduced in older age AD patients. Joo et al. found *in vitro* that ginsenoside Rg3 can promote microglia uptake, internalization and digestion of Aβ, which may be related to the expression of type A macrophage scavenger receptor (MSRA) (Joo and Lee, 2005). Further studies by Jang et al. determined that the uptake of Aβ42 by Rg3 is mediated by SRA and clathrin- and caveolae-dependent endocytosis, followed by accelerated degradation of Aβ42 via upregulation of NEP and IDE expression (Jang et al., 2015).

In addition, Li et al. reported that ginsenoside Rg1 not only reduced the accumulation of Aβ1-42 and phosphorylated (p)-Tau in AD models, but also activated the BDNF-TrkB pathway, improved long-term hippocampal potential enhancement and memory (Choi et al., 2016). In addition, Rg1 increased the expression of synaptic plasticity-related proteins, such as postsynaptic density-95 (PSD-95), synaptophysin and so on in the hippocampus via activating the mammalian rapamycin pathway, and improved behavior in elderly mice (Ong et al., 2015). These findings may suggest that Rg1 directly affects Aβ protein deposition, and it also controls various age-related proteins. A similar finding also confirmed that Rg1 reduced neuronal damage, cognitive impairment, and Aβ deposition by decreasing NADPH-oxidase2 (NOX2) activation in APP/PS1 mice, the most commonly used AD model (Zhang et al., 2021).

It has been reported that multiple protein kinase pathways are involved in α-secretase activation, such as extracellular signal-regulated kinase/mitogen-activated protein kinase (ERK/MAPK) and phosphatidylinositol-3 kinase (PI3K)/Akt, and protein kinase C(PKC) is also closely related to α -secretase activation (Zhu et al., 2001). Estrogen receptors (ERα and ERβ) are upstream of MARK and PI3K, and their phosphorylation activity mediates estrogen intracellular signal transduction. However, ginsenoside Rd has been found to have estrogen-like effects, and treatment with ginsenoside Rd can up-regulate the expression of ERα to enhance the α-secretase activity, thus accelerating the non-amyloid cleavage of APP processing, increasing the secretion and metabolism of sAPPα, and reducing the production of Aβ(19). But the improvement of these AD-related dysfunctions and activation of MAPK and PI3K pathways can be blocked by estrogen receptor antagonists (Yan et al., 2017). PI3K acts downstream of many receptors, among which the PI3K-Akt pathway is closely related to cell survival and regulates a variety of transcription factors (such as CREB, NF-κB) and a series of cellular functions, such as protein synthesis, apoptosis, cell differentiation and brain cognitive functions related to synaptic plasticity. Activation of the PI3K pathway was demonstrated to be non-essential for sAPPα production (Mei et al., 2010). In addition, the PI3K-Akt pathway regulates the expression and transport of many enzymes related to glucose metabolism (such as GSK-3 and Rheb) and glucose transporters (GLUTs) and participates in mitochondrial aerobic respiration, which has extensive significance for oxidative stress caused by free radicals.

### Inhibitory effects of ginsenosides on neurofibrillary tangles

NFTs are formed by hyperphosphorylation of tau (Liu et al., 2015a). Tau proteins are a group of microtubule-binding proteins, which are abundant in neurons and play an important role in neurite growth under physiological conditions such as promoting microtubule stability and axon transport (Johnson and Stoothoff, 2004), their phosphorylation is regulated by various proteases, such as glycogen synthase kinase 3 (GSK3), cyclin-dependent kinase-5 (CDK-5) and protein phosphatase-2A (PP2A). GSK3 (especially GSK3 β) plays a crucial part in the pathogenesis of AD, and inhibition of GSK3 can prevent Aβ aggregation and tau hyperphosphorylation (Hernandez et al., 2013). Calproteinase-mediated hydrolysis of cyclin-associated activating molecule p35 to p25 leads to dislocation of CDK-5, and then forms a stable complex with highly phosphorylated Tau protein, increasing the pathology of AD. Chen et al. demonstrated *in vitro* that Rb1 preconditioning could inhibit the transcription of CDK-5 and p25 in cortical neurons, stabilize intracellular calcium homeostasis and microtubule integrity (Chen et al., 2008), and thus weaken tau hyperphosphorylation. (Figure 3)

In addition, oxidative stress and mitochondrial abnormality are considered to be major factors in the formation of NFT in AD (Dumont et al., 2011; Zhao and Zhao, 2013), and the inflammatory environment may also activate Tau kinase to promote NFT formation (Glass et al., 2010). Okadaic acid (OA) is a selective inhibitor of protein serine/threonine phosphatases 1, 2A and 2B, causing tau hyperphosphorylation and neurofibromin accumulation, which is commonly used to mimic the AD symptoms of NFT injury. It was observed *in vivo* and *in vitro* experiments that pretreatment of ginsenoside Rd reduced OA-induced PP2A inactivation and inhibited tau hyperphosphorylation at Ser199/202, Ser396 and Ser404 (Li et al., 2011). Zhao H et al. demonstrated that aluminum exposure induces tau hyperphosphorylation, increases p-GSK and decreases PP2A levels in motor, sensory cortex and hippocampal, while ginsenoside Rb1 treatment reverses P-GSK3 and PP2A levels. Alleviating aluminum-induced toxicity significantly improved learning and memory (Zhao et al., 2013). Ginsenoside Rd also inhibits Aβ -induced Tau phosphorylation by changing the functional balance of GSK-3β and PP-2A (Li et al., 2013), but all of this was achieved under the condition of ginsenosides pretreatment. Further clinical trials are needed to clarify whether ginsenoside Rd has the potential as a drug to prevent the progression of AD.

## Ginsenosides associated with neuroprotective effects in AD

### Anti-oxidation effect of ginsenosides

Current researches along with these data show that oxidative instability is a critical initiating event in the etiology of AD. The brain has a high rate of oxygen metabolism and a relative lack of oxygen-free radical scavenging enzymes and antioxidant molecules, so it is easily affected by oxidative stress, resulting in inhibition of neurogenesis and obstruction of cognitive function. Brain aging is a high-risk factor for AD. During natural aging, all tissues and organs decline in function, and the accumulation of harmful substances such as free radicals can damage brain structure and function, particularly in the hippocampal region, which gives rise to neural stem cells (NSCs) that produce neurons and glial cells. Some studies have shown that oxidative damage caused by intracellular reactive oxygen species (ROS) is the main factor in inducing senescence of stem cells (Jung et al., 2014), and more and more evidence indicates that ROS accumulation plays a significant role in the development of AD. Therefore, a reasonable strategy to treat mitochondrial oxidative stress-related diseases is to enhance endogenous antioxidants. Some studies indicated that certain ginsenosides in intracellular cells can be used as free radical scavengers and increase the internal antioxidant enzyme, such as superoxide dismutase (SOD) and glutathione peroxidase (GSH-PX), which is related to activation of the Kelch-like epoxy chloropropane-related protein-1 (Keap1)/nuclear factor erythroid 2-related factor 2 (Nrf2)/antioxidant response element (ARE) signaling pathway (He et al., 2022).

NOX is the only known ROS-producing enzyme family, including several subtypes such as NOX1-5. NOX2 is mainly expressed in neurons, which can lead to age-related neuronal oxidative stress damage and brain function loss. Zhang H et al. found that ginsenosides Rg1 treatment significantly reduced the expression of NOX2 in H_2_O_2_-treated neurons, thus reducing ROS levels in the cortex and hippocampus, suggesting that Rg1 may alleviate cognitive dysfunction in AD by inhibiting NOX2-mediated neuronal oxidative stress (Zhang et al., 2021). Chen L et al. confirmed that ginsenoside Rg1 can enhance the activity of endogenous antioxidant enzymes, reduce oxidative stress to protect NSCs, promote brain neurogenesis and NSCs differentiation to neurons, not glial cells, and alleviate D-gal induced cognitive impairment in aging mice (Zhu et al., 2014; Chen et al., 2018). In addition, recent studies have shown that Akt and the mechanized targets of rapamycin (mTOR) are closely related to stem cell aging. Xie et al. demonstrated that ginsenoside Re can reduce oxidative damage and mitochondrial apoptosis induced by excessive ROS through activation of the PI3K/AKT signaling pathway (Xie et al., 2020). Ginsenoside Rg1 can also inhibit NSCs senescence by down-regulating Akt/mTOR signaling pathway (Chen et al., 2018).

### Anti-inflammatory effect of ginsenosides

Oxidative stress promotes inflammation and releases a variety of neurotoxic products and proinflammatory cytokines, such as IL-1β, IL-6 and TNF-α. The inflammasome is a multi-protein complex that plays an important role in the natural immune system and has been implicated in age-related neurodegenerative diseases. NLRP1 inflammasome is the main inflammasome in neurons, which can be activated by accumulated ROS. *In vitro* experiments confirmed that ginsenoside Rg1 inhibited the activation of NLRP1 inflammasome in hippocampal neurons by down-regulating NOX2 and reducing ROS production, thus inhibiting the aging and injury of neurons (Xu et al., 2019).

MAPK and NF-κB signaling pathways are key modulators of inflammatory responses. NF-κB is a transcription factor with multiple functions, which is closely related to pathophysiological processes such as inflammation and immune response and is involved in neurodegenerative diseases in recent studies (Wu et al., 2022). *In vivo* studies have shown that ginsenoside Rg1 inhibits nuclear NF-κB translocation from cytoplasm to the nucleus, which is required for activation, mediated by the receptor p65 for a low-affinity nerve growth factor (NGF).

Microglia are the innate immune effector cells of the central nervous system. When activated by endogenous or exogenous pathological injury, microglia will release BDNF and NT-3 and NGF to play a neuroprotective role as nutritional factors or release protective anti-inflammatory factors to alleviate nerve injury (Harry, 2013). However, over-activation of microglia will produce neurotoxicity. Microglia-mediated chronic neuroinflammation is involved in the pathological process of various neurodegenerative diseases such as AD. Liu J et al. found that after ginsenoside Rd treatment, the expression of NFκB p65 in cells was decreased, resulting in reduced production of traumatic pro-inflammatory factor, inhibition of NFκB transcriptional activity, inhibition of glial overactivation, and increased expression of protective factors (Liu et al., 2015b). Yang X et al. demonstrated that the neuroprotective effect of Rg1 is achieved by inhibiting the activation of MAPK and NF-κB signaling pathways and reducing the level of inflammatory factors (Yang et al., 2021). Bae et al. confirmed that ginsenoside Rh2 inhibited NO production in lipopolysaccharide (LPS) and interferon-gamma (IFN-γ) induced mouse microglia, which may be related to the decreased protein and mRNA expression of the iNOS gene. Further studies have proved that the anti-inflammatory effect of ginsenoside Rh2 seems to be related to the PKA pathway and activator protein 1 (AP-1) (Bae et al., 2006). In addition, ginsenoside Rb1 can play its anti-inflammatory function in AD by changing APP cleavage mode from amyloid to non-amyloid to prevent Aβ formation (Lin et al., 2019) and play a neuroprotective role.

### Anti-apoptosis effect of ginsenosides

It is well known that neuronal apoptosis is the main pathway of Aβ -induced neurotoxicity, and the prevention of Aβ -induced apoptosis is considered to be an important means of treating AD. It is noteworthy that mitochondria are at the central stage of human neurodegenerative diseases, and more and more evidence suggests that mitochondria are involved in Aβ -induced neuronal apoptosis. Apoptosis is a complex biological mechanism regulated by many signaling pathways. Multiple anti-apoptotic proteins (e.g., Bcl-2, Bcl-XL) and apoptotic proteins (e.g., Bax, Bad) in the Bcl-2 family, GSK-3β are involved. The activation of intrinsic apoptotic signals in mitochondria is determined by the balance between anti-apoptotic and pro-apoptotic proteins. Caspase-3 is considered to be the ultimate executor of apoptosis, which cleaves cytoskeleton and nuclear proteins.

Aβ can induce mitochondrial dysfunction and activate mitochondrial apoptosis pathways, which may be related to intracellular Ca^2+^ influx and the production of toxic substances such as ROS and H_2_O_2_, etc. in oxidative stress (Ponnusamy et al., 2009). The collapse of mitochondrial membrane potential (MMP) is an early landmark event that induces mitochondrial dysfunction, which can then lead to irreversible apoptosis. Zhou J et al. demonstrated *in vivo* and *in vitro* that ginsenoside Rd could block mitochondrial membrane potential dissipation and cytochrome C (Cyt C) release by activating the mitochondrial AKT/ERK signaling pathway and inhibiting mitochondrial apoptosis (Zhou et al., 2014). Besides, ginsenoside Re can block the loss of MMP, inhibit the release of Cyt C, and promote the production of ATP, thereby protecting mitochondrial function and protecting cells from Aβ -induced damage (Liu et al., 2019).

Annexinⅴ-FITC/PI double staining by Cui J et al. showed that ginsenoside Rg2 had a certain inhibitory effect on cell apoptosis induced by Aβ, but had no obvious inhibitory effect on cell necrosis (Cui et al., 2017). Wang Y et al. found that the inhibition of Rb1 on apoptosis may be realized by regulating the apoptotic signaling pathway. Specifically, Rb1 can down-regulate the expression of Bax, and caspase-3 and increase the expression of Bcl-2 in the hippocampus of rats, then inhibit neuronal apoptosis (Wang et al., 2018). Ginsenoside Rg1 acts as a ligand of the glucocorticoid receptor (GR) to activate the PI3K/Akt pathway that blocks Cyt C release and increases phosphorylation inhibition of the pro-apoptotic protein Bad, thereby inhibiting mitochondrial apoptosis (Cho, 2012). Rg1 was also found to be a significant anti-apoptotic molecule, blocking the Caspase-dependent signaling cascade in Jurkat T lymphoma cells, and also increasing humoral and cell-mediated immune responses (Li et al., 2014). Zhong et al. found that the anti-apoptotic effect of ginsenoside Rg1 may be related to the restoration of the Fibroblast growth factor (FGF2)/Akt signaling pathway (Zhong et al., 2020). Endoplasmic reticulum (ER) stress has been reported to be associated with neurodegenerative diseases. Ginsenoside Rb1 can protect nerve cells from high glucose-induced apoptosis by inhibiting the activation of ER stress-related proteins (Liu et al., 2013). NMDAR is an ionic glutamate receptor that is closely associated with a variety of neurological diseases. When it is overactivated, such as induced by excessive glutamate, it can lead to Ca^2+^ overload and neuroexcitatory toxicity, resulting in neuronal necrosis or apoptosis. Recent studies have found that ginsenoside Rd, reduces the phosphorylation of NR2b subunit mediated by death-related protein kinase 1 (DAPK1) by reducing CaN, a phosphatase associated with Ca^2+^ regulation, activity, thus inhibiting Ca^2+^ influx and excitatory toxicity induced by NMDAR (Zhang et al., 2020); (Zhang et al., 2012). In addition, Li X et al. demonstrated that ginsenoside Rd can also block glutamate-induced Ca^2+^ entry from voltage-independent Ca^2+^ channels ROCC and SOCC (Li et al., 2010) and protect neurons from neurotoxic damage, which has the potential as a new Ca^2+^ channel blocker.

**FIGURE 4:**
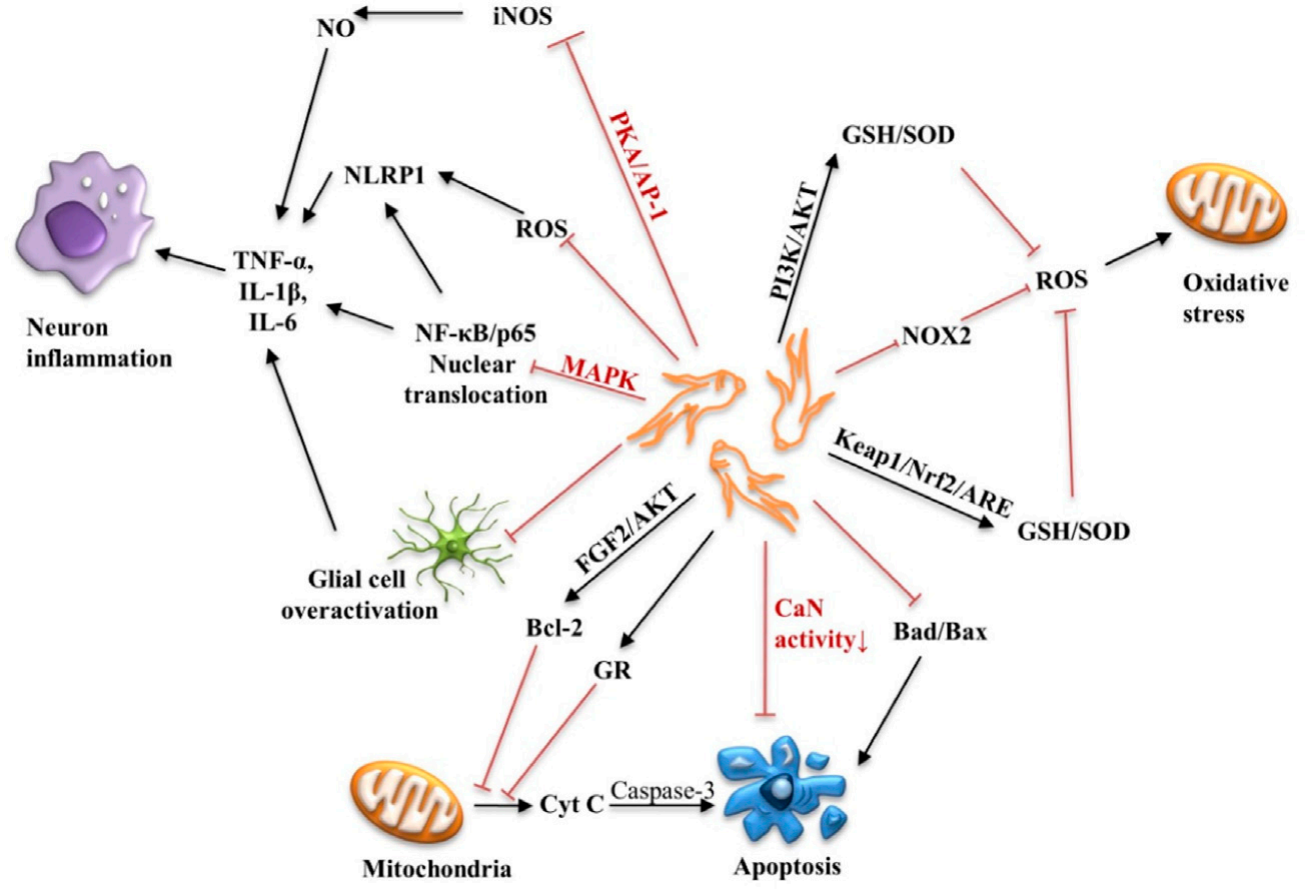
Schematic diagram of the neuroprotective mechanism of ginsenosides. PKA, protein kinase A; AP-1, activator protein 1; MAPK, mitogen-activated protein kinase; NF-κB, nuclear factor Kappa B; TNF-α, Tumor necrosis factor α; IL-1β, Interleukin-1β; IL-6, Interleukin-6; FGF, fibroblast growth factor; GR, glucocorticoid receptor; cyt C, cytochrome C; Keap1, Kelch-like epoxy chloropropane-related protein-1; Nrf2, nuclear factor erythroid 2-related factor 2; ARE, antioxidant response element; NOX2, NADPH-Oxidase 2; PI3K, phosphoinositol 3 kinase; SOD, superoxide dismutase; GSH, glutathione.

Apoptosis of neurons or nerve cells is an important pathological factor that cannot be ignored in the development of AD. Aβ deposition, NFT formation, oxidative stress and neuroinflammation can all lead to the death of neurons. Therefore, reducing oxidative stress, neuroinflammation, and apoptosis can help delay the progression and neurodegenerative changes of AD. The neuroprotective mechanism of ginsenosides is shown in Figure 4.

## Ginsenosides in clinical application

Neurodegenerative diseases are complex treatments in which the brain is surrounded by a highly protective blood-brain barrier (BBB) that restricts the entry of non-brain-specific substances, thus limiting the clinical use of many drugs. In the past 30 years, the United States Food and Drug Administration (FDA) has approved five drugs for the treatment of AD, including NMDA receptor antagonists and acetylcholinesterase inhibitors (AChEI), but all of them are symptomatic and cannot prevent or slow the loss of neurons and abnormal brain function. Drugs that affect the course of the disease are still in the preliminary stage of research. The latest figures show that FDA announced in June 2021 that it approved a biologics licensing (BLA) application for aducanumab (Aduhelm), an Aβ antibody from Biogen, for the treatment of patients with early-stage AD (Dhillon, 2021). Aducanumab is the first new drug approved by the FDA for the treatment of AD since 2003. The drug has been shown to work well in patients with mild cognitive impairment or mild dementia in clinical trials, and there is no safety or efficacy data for starting treatment at an earlier or later stage of the disease (Sabbagh and Cummings, 2021), which is part of the drug’s controversy.

Although ginsenosides have made substantial progress in delaying the process of AD, the limitation of its research lies in that these results are reflected in animal and cell culture studies, and few clinical trials on the effect of ginseng on AD have been completed. The clinical trial data collected at present are collected and sorted out in Table 2. In open-label clinical trials, Heo et al. treated AD patients with low or high concentrations of KRG for 12 weeks, and assessed changes in cognitive and functional performance using the Alzheimer’s Disease Assessment Scale (ADAS), the Korean version of mini Mental State Examination (K-MMSE), and the Clinical Dementia Assessment Scale (CDR). The KRG is made by steaming fresh and unpeeled Panax ginseng. The ginsenosides contained in KRG include Rb1, Rb2, Rc, Rd, Re, Rf, Rg1, Rg2, Rg3, Rh1, and Rh2, accounting for 8.54% of the total proportion (Heo et al., 2011). Patients with AD were considered older than 50 years, had a baseline score of 10–26 on the SIMPLE Mental status Test, and had no history of mental illness, epilepsy, or cognitive impairment due to stroke, hypoxic brain injury, brain tumor, infection, or antidepressant or psychotropic medication. Trial results showed significant improvement in ADAS and CDR in the high-dose KRG group after 12 weeks of KRG treatment compared with the control group (Heo et al., 2008). Later, the same team conducted a small trial of 14 AD patients. K-MMSE and the Frontal Assessment Battery (FAB) were used to assess cognitive function and quantitative electroencephalogram (EEG) changes before and after KRG treatment for 12 weeks. The FAB score was significantly improved after treatment. In addition, the effect of KRG on frontal lobe function in AD patients is associated with an increase in relative α power (Heo et al., 2016). Lee et al. used the MMSE and ADAS to monitor cognitive changes at 12 weeks of ginseng treatment and 12 weeks of withdrawal. The results showed that ginseng treatment improved ADAS and MMSE scores, but with discontinuation of treatment, both scores gradually decreased back to the control level. Trials suggest that ginseng has clinical efficacy in the cognitive performance of patients with AD (Lee et al., 2008). Heo et al. subsequently recruited subjects for treatment and follow-up for up to 2 years, during which ADAS and K-MMSE scores did not decrease, suggesting that KRG has a long-term effect on AD, lasting at least 2 years (Heo et al., 2011). In addition, there were no adverse reactions in all clinical studies, which preliminarily suggest that ginseng treatment is safe, better tolerated in AD patients, and has a positive effect on the cognition of AD patients.

**TABLE 2:** Summary of clinical trials of ginseng intervention in AD.

Medicine	Sample	Treatment group	Evaluative criteria	Results	Ref
Korean red ginseng (KRG)	Sixty-one AD patients, aged 50–80 years	Low-dose (4.5 g/day, n = 15), high-dose (9 g/day, *n* = 15), control (*n* = 31)	ADAS, K-MMSE, CDR	Patients in the high-dose KRG group showed significant improvement in ADAS and CDR after 12 weeks of KRG treatment compared with the control group	Heo et al. (2008)
Korean red ginseng (KRG)	Fourteen patients with AD (mean age, 74.93 years; 11 women and 3 men)	Patients treated with KRG (4.5 g per day) for 12 weeks	K-MMSE, FAB	The FAB score improved significantly	Heo et al. (2016)
Panax ginseng powder	AD patients	Ginseng group (4.5bg/d for 12 weeks, *n* = 58), control group (*n* = 39)	MMSE, ADAS	Ginseng treatment improved ADAS and MMSE scores, but with the withdrawal of treatment, both scores gradually decreased back to the control group level.	Lee et al. (2008)
Korean red ginseng (KRG)	Sixty-one AD patients between the ages of 50 and 80	Low-dose (4.5 g/day, *n* = 15), high-dose (9 g/day, *n* = 15), control (*n* = 31)	ADAS, K-MMSE, CDR	KRG treatment did not show a decrease in scores measured by ADAS-COG and K-MMSE over 2 years.	Heo et al. (2011)

Despite ginsenosides having shown great therapeutic potential for the treatment of AD, there are still some shortcomings that cannot be ignored. Pharmacokinetic studies show that the oral bioavailability of ginsenoside Rg1 is poor, only 1.9%–20.0% (Xue et al., 2016). Exploring new delivery modalities, such as direct delivery of the drug to the brain via the intranasal route, with advantages of high bioavailability, avoidable gastrointestinal irritation, degradation and one-time metabolism of the drug in the gastrointestinal tract and liver, maybe a promising strategy (Long et al., 2020). In addition, subcutaneous, intraperitoneal, and inner ear administration provides better BBB permeability than oral administration. On the other hand, changing drug delivery is also a strategy to improve drug pharmacokinetic characteristics. In the past decades, various nanoparticles were developed, including polymer coupling material, polymer NPs, and aliphatic carriers, they package take ginsenosides through BBB delivery to the brain (Gupta et al., 2019). NPs have been shown to improve the physicochemical properties of Aβ inhibitors, such as targeting small molecules, proteins and peptides, and allow them to be delivered to the brain via BBB, improving bioavailability (Shukla et al., 2021). For example, Nao-Qing microemulsion is a preparation composed of P. ginseng roots and other Chinese herbs, which contain multiple active ingredients including ginsenoside Rg1 (Li et al., 2015). Compared with oral administration, intranasal administration significantly promoted drug absorption. Nanoencapsulation technology can not only effectively improve the pharmacokinetic characteristics of ginsenosides, but also improve the targeting, specificity, stability and safety of drugs, which is of great significance for the diagnosis and treatment of AD.

## Conclusion and Perspective

From the perspective of the current study on the pathogenesis of AD, its pathogenic factors are very complex, which brings some difficulties to the research and development of AD treatment drugs but also brings opportunities for the study of the role of natural drugs in AD. The failure of many clinical trials suggests that treating AD with a single target is difficult, while multi-target drugs and cocktail combination drugs may be an important direction of AD drug development in the future. China has unique advantages in the research of natural products, many of which have multi-target pharmacological activity. As a famous natural Chinese herbal medicine, ginseng, its active ingredient (especially ginsenosides), has shown good neuroprotective effects through various mechanisms without side effects and has been proved to be beneficial to the prevention of neurodegenerative diseases for many years. In this review, we systematically and comprehensively summarize recent studies on the effects of ginsenosides on cognitive and memory dysfunction in AD patients or animal models, demonstrating that ginseng can block or improve the pathological process at different stages of AD by regulating a variety of signaling molecules and pathways, including inhibition of Aβ production and accumulation, tau hyperphosphorylation, inhibition of oxidative stress, neuroinflammation, apoptosis and mitochondrial dysfunction. In addition, from the perspective of pharmacokinetics, it is a new strategy that changes the delivery mode or develops new drug delivery vectors to improve the clinical application value of ginsenosides. Overall, this review provides a deeper insight into the understanding of the application of ginsenosides and its further development may create a new situation for the treatment of AD.
